# Aberrant Activation of ERK/FOXM1 Signaling Cascade Triggers the Cell Migration/Invasion in Ovarian Cancer Cells

**DOI:** 10.1371/journal.pone.0023790

**Published:** 2011-08-17

**Authors:** Gabriel T. M. Lok, David W. Chan, Vincent W. S. Liu, Winnie W. Y. Hui, Thomas H. Y. Leung, K. M. Yao, Hextan Y. S. Ngan

**Affiliations:** 1 Department of Obstetrics and Gynaecology, LKS Faculty of Medicine, The University of Hong Kong, Hong Kong SAR, People's Republic of China; 2 Department of Biochemistry, LKS Faculty of Medicine, The University of Hong Kong, Hong Kong SAR, People's Republic of China; University of Michigan, United States of America

## Abstract

Forkhead box M1 (FOXM1) is a proliferation-associated transcription factor essential for cell cycle progression. Numerous studies have documented that FOXM1 has multiple functions in tumorigenesis and its elevated levels are frequently associated with cancer progression. Here, we characterized the role of ERK/FOXM1 signaling in mediating the metastatic potential of ovarian cancer cells. Immunohistochemical (IHC), immunoblotting and semi-quantitative RT-PCR analyses found that both phospho-ERK and FOXM1 were frequently upregulated in ovarian cancers. Intriguingly, the overexpressed phospho-ERK (*p*<0.001) and FOXM1 (*p*<0.001) were significantly correlated to high-grade ovarian tumors with aggressive behavior such as metastasized lymph node (5 out of 6). Moreover, the expressions of phospho-ERK and FOXM1 had significantly positive correlation (*p*<0.001). Functionally, ectopic expression of FOXM1B remarkably enhanced cell migration/invasion, while FOXM1C not only increased cell proliferation but also promoted cell migration/invasion. Conversely, inhibition of FOXM1 expression by either thiostrepton or U0126 could significantly impair FOXM1 mediated oncogenic capacities. However, the down-regulation of FOXM1 by either thiostrepton or U0126 required the presence of p53 in ovarian cancer cells. Collectively, our data suggest that over-expression of FOXM1 might stem from the constitutively active ERK which confers the metastatic capabilities to ovarian cancer cells. The impairment of metastatic potential of cancer cells by FOXM1 inhibitors underscores its therapeutic value in advanced ovarian tumors.

## Introduction

Ovarian cancer is one of the most lethal gynecologic malignancies worldwide. Due to the non-specific symptoms, most of ovarian cancer cases are presented with advanced stage disease and associate with high mortality rate [1]. In the advanced ovarian cancer, tumor cells are highly invasive and the subsequent cancer metastasis leads to death [2]. Both cell migration and invasion contribute the metastatic ability of the tumor cells and the genetic mechanism that regulates metastasis remains largely unknown. Therefore, it is of paramount importance to identify molecular mediators conferring the metastatic potential to ovarian cancer cells that may be used as tumor markers in predicting risk of ovarian cancer progression.

Forkhead Box M1 (FOXM1) is a typical transcription factor that belongs to the Forkhead Box family [3]. FOXM1 is well-known for its critical role in cell cycle progression by regulating the G1/S and G2/M phases of cell cycle transition and ensuring a proper execution of mitotic cell division [3], [4], [5]. However, mounting evidence supports a link between aberrant expression of FOXM1 and human carcinomas such as pancreatic cancer, basal cell carcinomas, glioblastomas and cervical cancer [6], [7], [8], [9]. More importantly, the increased expression of FOXM1 has remarkable correlation with the progressive stages of numerous human cancers [6], [9], [10], [11], [12]. Hence, FOXM1 not only promotes tumorigenesis by endowing proliferative capacity and leading to uncontrolled cell division at the early period of cancer development but also enhances other tumorigenic behaviors in other stages of cancer development [12], [13], [14]. To date, several lines of evidence have shown that FOXM1 could enhance angiogenesis in glioma cells [15], anchorage-independent growth ability in cervical cancer cells [6], tumor growth ability of glioma cells [7] and metastasis of hepatocellular carcinomas cells in nude mice [16], implying the diverse roles of FOXM1 in tumorigenesis. Recently, the Cancer Genome Atlas Research Network has used a probabilistic graphical model (PARADIGM) to prove that FOXM1 signaling is critically associated with serous ovarian cancer pathophysiology [17]. However, the expression status and functional roles of FOXM1 in ovarian cancer, especially in cell migration/invasion are largely speculative. This highlights an urgent need to delineate the molecular mechanism underlying the cancer progression so as to increase the survival rate of patients with ovarian cancer.

Constitutive activation of ERK signaling is usually linked with neoplastic transformation such as uncontrolled cell growth by stimulating cell survival pathway and increasing cell motility [18], [19], [20], [21]. Previous studies have reported that ERK acts upstream of FOXM1 and the phosphorylation of FOXM1 by ERK are required for the nucleus translocation and subsequent transactivation [22]. In addition, FOXM1 has been shown to be one of the ERK effectors in human hepatocellular carcinoma and breast cancer [23], [24]. However, the function of ERK/FOXM1 signaling cascade in regulating cell migration/invasions has not yet been clearly elucidated.

In this study, we firstly analyzed the expression status and clinicopathological correlation of FOXM1 in ovarian cancer. Several tumorigenic assays were conducted in ovarian cancer cell models using gain- and loss-of function of FOXM1 through enforced expression of FOXM1 or inhibition of endogenous FOXM1 by thiostrepton or U0126. We also gave the data showing the importance of p53 status in the use of FOXM1 inhibitors. Moreover, we provided the first *in vitro* evidence of ERK/FOXM1 signaling cascade in promoting cell migration/invasion in ovarian cancer cells. This confers an encouraging target in combating advanced ovarian cancer.

## Results

### FOXM1 is over-expressed and correlates with pERK expression and high-grade subtype of ovarian cancer

**Figure 1:**
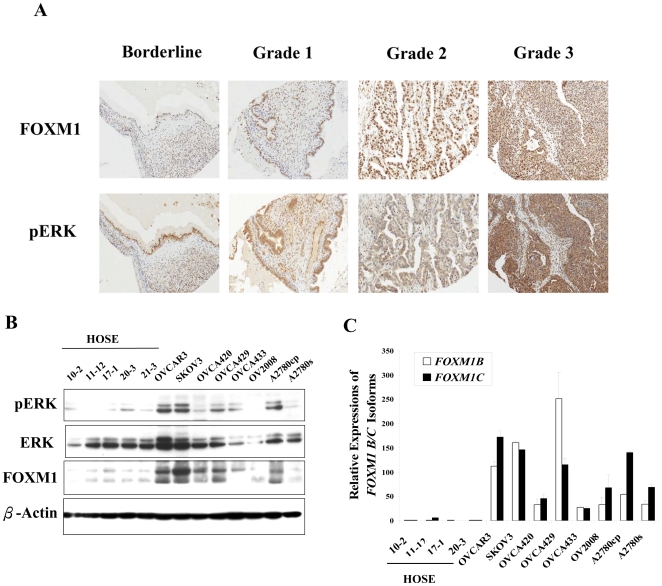
FOXM1 and pERK are over-expressed in high-grade ovarian tumor and correlate to each other in ovarian tumors and cancer cell lines. (A) Representative IHC showed the immunoreactivities of FOXM1 and pERK in borderline, Grade 1, Grade 2 and Grade 3 tumor on a ovarian cancer tissue array (OVC1021) (x20). Increasing staining of both FOXM1 and pERK was observed along with the progression of cancer which implicates their potential roles in tumorigenesis. (B) Western blotting showed the expressions of pERK, ERK and FOXM1 in HOSEs and ovarian cancer cell lines. (C) Real time QPCR analysis revealed the expressions of FOXM1 isoforms; *FOXM1B* and *FOXM1C* in HOSEs and ovarian cancer cell lines. *FOXM1A* was neglected in this experiment because it was reported as being transcriptionally inactive.

To study the significance of FOXM1 expression in ovarian cancer development, IHC analysis on a commercial tissue array of 97 ovarian tissue samples, including 2 normal, 2 benign, 1 borderline cystademoma and 96 malignant tumor samples was conducted. High FOXM1 expression was significantly correlated with high-grade tumors (Grade 3) (*P*<0.001), in which 73.33% of Grade 3 tumors cases exhibited over-expression of FOXM1 whereas 68% of Grades 1 and 2 tumors cases showed low expression of FOXM1 (Table 1). Regarding the tumor subtype, the expression of FOXM1 was highly correlated with endometriod adenocarcinoma (*P*<0.05), in which 74.07% of cases demonstrated over-expression of FOXM1 (Table 1). Additionally, 5 out of 6 cases with lymph node metastasis showed over-expression of FOXM1 (data not shown). Despite the limited sample size, it could be a reference for an association between FOXM1 and distant metastasis. Besides, a remarkable correlation was detected between the up-regulation of both FOXM1 and pERK (*P*<0.001) (Table 1). Similar to FOXM1, a significant correlation was detected between the expression of pERK and high-grade tumors (*P*<0.001), in which 66.67% of Grade 3 tumors showed high expression of pERK (Table 2). Concordant weak to very strong staining of pERK and FOXM1 were also observed from borderline cystademoma to Grade 3 tumor, implicating the importance of the two proteins in tumor progression of ovarian cancer (Fig. 1A).

**Table 1:** Clinical-pathological analysis of FOXM1expression.

		FOXM1 expression		
Parameters	n( = 96)	≤150	>150	*p*
**Grade**				
1 and 2	50	34 (68%)	16 (32%)	
3	45	12 (26.67%)	33 (73.33%)	<0.001*
**Stage**				
Early	48	24 (50%)	24 (50%)	
Late	48	22 (45.83%)	26 (54.17%)	1
**Subtypes**				
Endometriod Adenocarcinoma	27	7 (25.93%)	20 (74.07%)	
Others	69	42 (60.87%)	27 (39.13%)	0.003*
Musinous Cystadenocarcinoma	21	13 (61.9%)	8 (38.1%)	
Others	75	35 (46.67%)	40 (53.33%)	0.324
Serous Cystadenocarcinoma	49	28 (57.14%)	21 (42.86%)	
Others	47	21 (44.68%)	26 (55.32%)	0.307
**pERK**				
Low Expression	67	46 (68.66%)	21 (31.34%)	
High Expression	34	7 (20.59%)	27 (79.41)	<0.001*

**Table 2:** Clinical-pathological analysis of phospho-ERK expression.

		pERK expression		
Parameters	n ( = 96)	≤150	>150	*p*
**Grade**				
1 and 2	51	46 (90.2%)	5 (9.8%)	
3	45	15 (33.33%)	30 (66.67%)	<0.001*
**Stage**				
Early	47	31 (66%)	16 (34%)	
Late	49	29 (59.18%)	20 (40.82%)	0.675

For the cell lines study, the expressions of pERK and FOXM1 also demonstrated a good correlation in a panel of ovarian cancer and HOSEs cell lines. Both proteins were highly expressed in cancerous cell lines, especially in OVCAR3, SKOV3, OVCA429 and A2780cp, whereas they showed lower expressions in HOSE cell lines (Fig. 1B). Similarly, real time QPCR analysis using specific primers according to previous report [12] revealed that both *FOXM1B* (27 to 250-fold) and *FOXM1C* (25 to 172-fold) were up-regulated in ovarian cancer cell lines as compared with HOSE cell lines (Fig.1C). This result was consistent to the findings of western blot analysis and also showed that there was no difference of the expression patterns between two isoforms in ovarian cancer cells. Collectively, these data suggest that both ERK activity and FOXM1 expression are up-regulated and positively correlated in ovarian cancers, especially in aggressive high-grade tumors, which implicates sharing of a common pathway in ovarian cancer progression.

### FOXM1 promotes cell proliferation and cell migration/invasion

**Figure 2:**
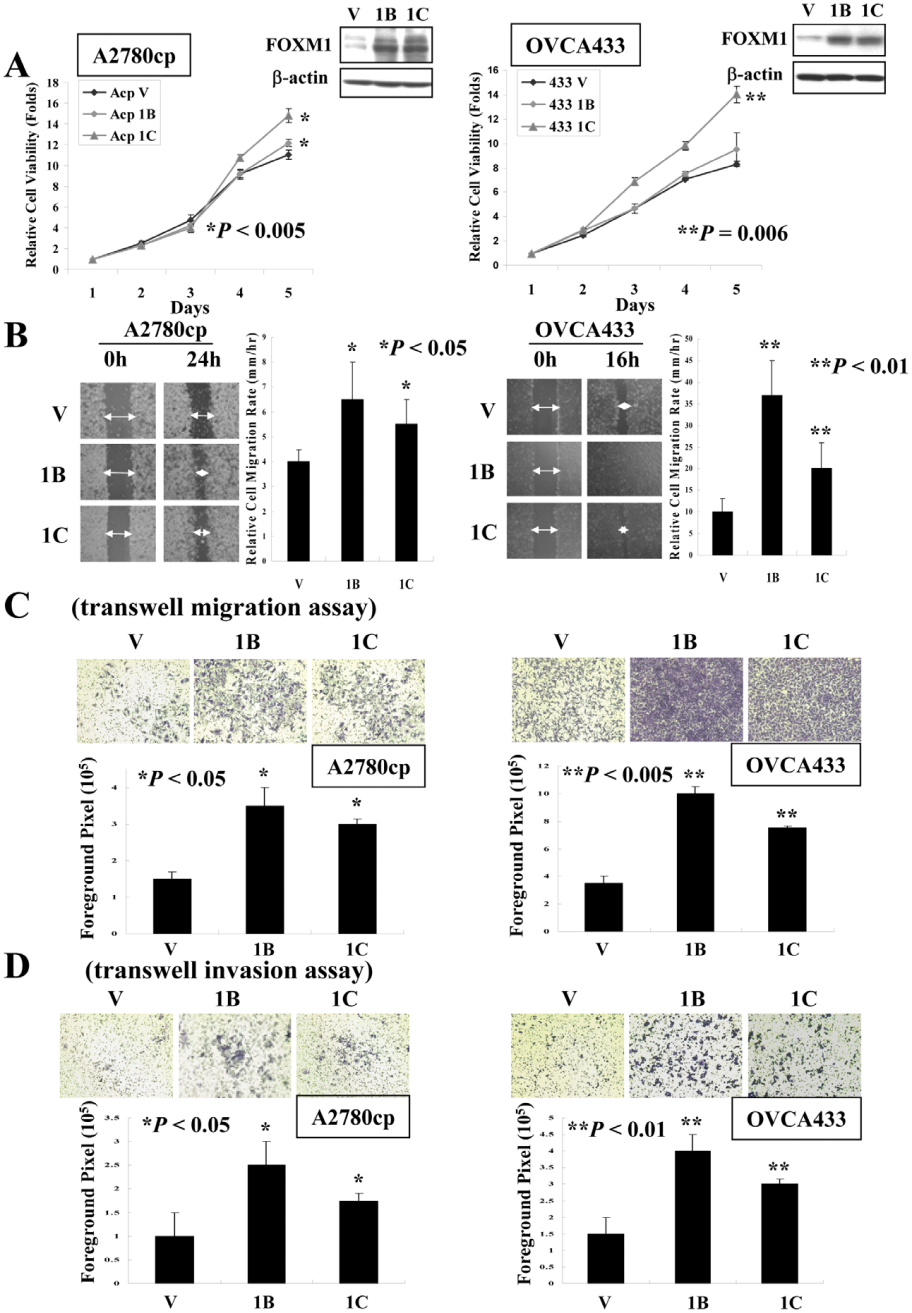
Up-regulation of FOXM1B or FOXM1C isoforms induces cell proliferation, migration and cell invasion in ovarian cancer cells. (A) XTT cell proliferation assay demonstrated that FOXM1C could remarkably increase cell proliferation of in A2780cp (*P<0.005) and OVCA433 (**P = 0.006) ovarian cancer cells, while FOXM1B just had slightly effect. Western blotting showed the expressions of FOXM1B (1B) and FOXM1C (1C) in A2780cp and OVCA433 cells by using anti-FOXM1 (K19). The empty vector was used as negative control (V). (B) Wound healing assay showed faster wound closure rate of FOXM1B or FOXM1C ectopic expressing A2780cp (*P<0.05) and OVCA433 (**P<0.01) cells as compared with vector control (*P<0.05). (C) Transwell migration assay and the bar chart showed higher migratory rate in FOXM1B and FOXM1C ectopic expressing cells of A2780cp (*P<0.05) and OVCA433 (*P<0.005) as compared with their vector controls (V). (D) Transwell cell invasion assay and the bar chart showed higher invasion rate through Matrigel-coated membrane in FOXM1B and FOXM1C ectopic expressing cells of A2780cp (*P<0.05) and OVCA433 (*P<0.01) as compared with their vector controls (V). The above experiments were performed thrice independently and the result was plotted.

Given that high-grade tumors were highly proliferative and metastatic, we reasoned that FOXM1 plays a certain role in regulating cell motility and invasion. To probe the oncogenic roles of FOXM1 in ovarian cancer, we assessed the functional effects of two active FOXM1 isoforms, FOXM1B and FOXM1C, on A2780cp and OVCA433 cells (Fig. 2A). Consistent with previous reports [14], [25], FOXM1C could increase the cell proliferation capacity in A2780cp (*P*<0.005) and OVCA433 (*P* = 0.006) ovarian cancer cells, while FOXM1B just showed slight increased effect (Fig. 2A). On the other hand, ectopic expression of FOXM1B and FOXM1C could promote faster wound closure in A2780cp and OVCA433 ovarian cancer cells by wound healing assay (Fig. 2B). By transwell migration/invasion assays, introduction of FOXM1B and FOXM1C also showed a significant increased cell migration (*P*<0.05 in A2780cp and *P*<0.005 in OVCA433) (Fig. 2C) and cell invasion (*P*<0.05 in A2780cp and *P*<0.01 in OVCA433) (Fig. 2D) abilities in A2780cp and OVCA433 cells compared with the vector controls. Interestingly, FOXM1B had relatively stronger influence in promoting cell migration and invasion as compared with FOXM1C. Taken together, these data confer that FOXM1 isoforms could differentially promote cell proliferation, migration/invasion in ovarian cancer cells.

### Effective inhibition of FOXM1 expression by thiostrepton requires p53

**Figure 3:**
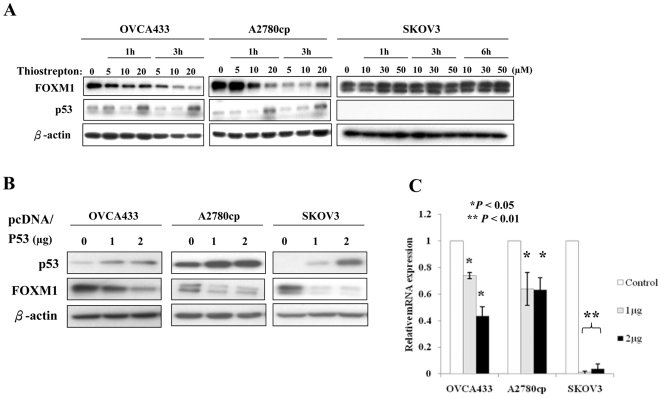
p53 is essential for the thiostrepton-mediated down-regulation of FOXM1. (A) Western blotting revealed the down-regulation of FOXM1 and up-regulation of p53 in ovarian cancer cell lines; OVCA433 and A2780cp, but not in SKOV3 upon treatment of thiostrepton under various dosages and time intervals. (B) Western blotting showed that transient transfection with p53 could suppress FOXM1 expression in ovarian cancer cells (OVCA433, A2780cp and SKOV3). (C) Real time PCR indicated down-regulation of *FOXM1* transcripts upon the transient transfection of p53.

The thiazole antibiotic thiostrepton can specifically inhibit the expression of FOXM1 at gene promoter level in breast cancer cell lines [26], and therefore it was used to deplete the expression of FOXM1 in ovarian cancer cells. Upon thiostrepton treatment, we observed different responses of FOXM1 in ovarian cancer cell lines carrying different p53 genotypic statuses: OVCA433 (p53 wild-type), A2780cp (p53 mutant) and SKOV3 (p53 deleted). OVCA433 and A2780cp cells showed a pronounced drop of FOXM1 expression in a time- and dosage-dependent manner, as well as an increased expression of p53 at higher dose (20 µM) of thiostrepton (Fig. 3A). On the other hand, SKOV3 was resistant to thiostrepton and FOXM1 expression sustained at 10, 30 and 50 µM. These findings suggest that p53 might be involved in the thiostrepton acting mechanism. Considering the very opposite roles of p53 and FOXM1 in cell cycle regulation and the negative impact of p53 on FOXM1 expression shown in previous micro-array result [5], [27], [28], [29], [30], we assessed the expression of FOXM1 upon alteration of p53 expression in ovarian cancer cells. Transient transfection of p53 remarkably reduced FOXM1 not only in protein level (Fig. 3B) but also in mRNA level (Fig. 3C) in OVCA433, A2780cp and SKOV3. These results indicate that p53 negatively regulates both mRNA and protein levels of FOXM1 and p53 is essential for thiostrepton to exert the inhibition on FOXM1 expression in ovarian cancer cells.

### Down-regulation of FOXM1 impairs cell migration/invasion

**Figure 4:**
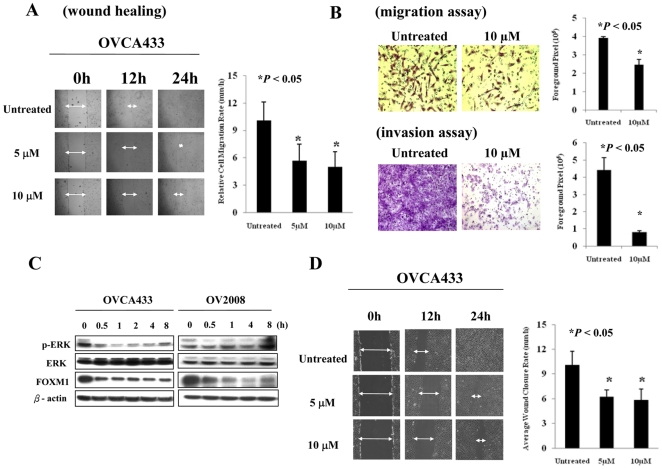
ERK activity requires FOXM1in regulating cell migration of ovarian cancer cells. (A) Wound healing assay showed lower wound closure rate of thiostrepton-treated OVCA433 cells as compared with untreated control (*P<0.05). (B) Transwell migration assay and the bar chart showed significantly lower number of migratory cells through Matrigel-coated membrane in FOXM1-depleted OVCA433 cells than the control (*P<0.05) (*upper*). Transwell cell invasion assay and the bar chart showed lower invasion rate in FOXM1-depleted OVCA433 cells following thiostrepton treatment when compared with the control (*P<0.05) (*lower*). (C) Western blotting revealed the down-regulation of FOXM1 in ovarian cancer cell lines (OVCA433 and OV2008) upon treatment with U0126 for various time intervals. (D) Wound healing assay demonstrated lower wound closure rate of U0126-treated OVCA433 cells as compared with untreated control (*P<0.05). Three independent experiments were performed and the result was plotted.

We hypothesized that thiostrepton can repress the ovarian cancer cell migration/invasion by inhibiting the expression of FOXM1. Since thiostrepton could induce apoptotic effect on cancer cells [26], XTT assays were performed thrice to exclude the factor of reduced cell viability which may affect the accuracy of using wound closure time for cell migration evaluation. Indeed, the cell viability of the above cell lines showed no significant changes upon treatment of the dosage range of 0 to 10 µM thiostrepton (Fig. S1). Upon treatment with thiostrepton, OVCA433 cells showed an obvious repression of cell migration, with ∼50% decrease of wound closure rate as compared with the untreated control in wound healing assay (*P*<0.05) (Fig. 4A). Similar inhibitory effect on cell migration was observed in A2780cp cells (data not shown). However, treatment on SKOV3 showed no significant influence on cell migratory inhibition (Fig. S2). Furthermore, treatment of 10 µM thiostrepton on OVCA433 also dramatically reduced approximately 37% cell migration (*P*<0.05), and 75% cell invasion (*P*<0.05) abilities as compared with controls in Matrigel assay (Fig. 4B). Taken together, these data suggest that FOXM1 significantly regulates cell proliferation and cell migration/invasion in ovarian cancer cells.

### ERK activity regulates cell migration mediated by FOXM1 in ovarian cancer cells

As the above study implied the plausible association of ERK activity and FOXM1 expression (Fig. 1A and 1B), it is interesting to investigate the underlying molecular mechanism. OVCA433 and OV2008 cells were treated with the MEK inhibitor U0126 and the levels of pERK and FOXM1 were evaluated at different time points by western blot analysis. A remarkable decrease of pERK level was observed in both cell lines as early as 30 minutes and there was a concomitant reduction of FOXM1 expression (Fig. 4C). Besides, the inhibition of FOXM1 expression sustained as long as the ERK activity remained suppressed (Fig. 4C). Since ERK lies up-stream of FOXM1 [23], [24], we reasoned that inhibition of ERK activity should abrogate cell migration of ovarian cancer cells through down-regulating FOXM1. Indeed, using wound healing assay, OVCA433 cells treated with U0126 showed a significant increase of wound closure time (2-fold more) compared with the control (*P*<0.05) (Fig. 4D). On the other hand, there was no reduction of FOXM1 expression and cell motility in SKOV3 cells (p53 deleted) upon treatment of U0126 (Fig. S3), indicating that the presence of p53 is required for U0126 or thiostrepton mediated FOXM1 inhibition. Taken together, these findings support an essential role of FOXM1 in mediating the effect of ERK on cell migration in ovarian cancer cells.

### MMP-9 and uPAR are FOXM1 downstream targets regulating cell migration/invasion

**Figure 5:**
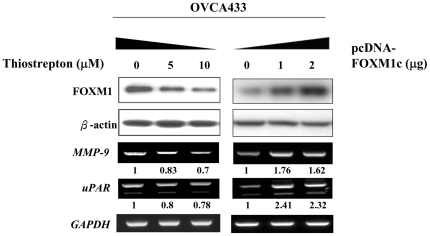
FOXM1 regulates the cell migration/invasion through up-regulation of *MMP-9* and *uPAR* expressions. Semi-quantitative RT-PCR showed that inhibition of FOXM1 expression by thiostrepton (5 and 10 µM) in OVCA433 caused a decrease of *MMP-9* and *uPAR* expressions (*left*), whereas transiently transfection with FOXM1 expression plasmid (1 and 2 µg) in OVCA433 showed an increase of *MMP-9* and *uPAR* expression (*right*). The numerical value under each panel referred to the mRNA expression level relative to the control.

Extracellular proteases like metalloprotease-9 (MMP-9) and urokinase receptor (uPAR) are markers to the acquisition of the metastatic capability [2]. Hence, we examined the levels of *MMP-9* and *uPAR* to confirm the effect of FOXM1 on cell motility and invasion. By suppressing FOXM1 expression with 5 and 10 µM thiostrepton in OVCA433, the levels of both *MMP-9* and *uPAR* were reduced by about 20 to 30% (Fig. 5). Conversely, transient over-expression of FOXM1 increased *MMP-9* and *uPAR* levels by ∼1.7-fold and ∼2.4-fold, respectively (Fig. 5). These data suggest that FOXM1 regulates the cell migration/invasion through transcriptionally up-regulating the expressions of *MMP-9* and *uPAR*.

## Discussion

In this study, we have showed that FOXM1 was aberrantly up-regulated in ovarian cancer, particularly in high-grade sub-type. Similarly, increased phospho-ERK in concomitant with FOXM1 was significantly associated with high-grade ovarian cancer. We thus proposed that FOXM1 overexpression might stem from constitutively activated ERK signaling that contributes to the metastatic capability in cancer cells. Our study indicates that the ERK/FOXM1 signaling cascade may be a promising target for therapeutic intervention in ovarian cancer.

We and other groups have previously reported that FOXM1 is frequently deregulated in a variety of human cancers [31]. Importantly, the increased FOXM1 expression was associated with the tumor progression of human cancers [6], [9], [10], [11], [12]. However, the functional roles of FOXM1 in ovarian cancer remain largely unexplored. Here, we showed that both mRNA and protein levels of FOXM1 were up-regulated in ovarian cancer tissues and cell lines. FOXM1 overexpression was significantly correlated with high-grade ovarian tumors, indicating that FOXM1 may play an oncogenic role in ovarian cancer, especially in the high-grade subtype. We therefore explored the effect of FOXM1 in cell migration/invasion which is a common tumorigenic scenario observed in aggressive high-grade tumors.

Both transcriptionally active splice variants of FOXM1; *FOXM1B* and *FOXM1C* are shown to be elevated in numerous human cancers. Particularly, FOXM1B was demonstrated to promote angiogenesis, tumor growth of glioma cells and metastasis of HCC in the absence of Arf while FOXM1C was found to enhance cell growth and anchorage-independent ability of cervical cancer cells [6], [7], [15], [16]. Here, enforced expression of either FOXM1B or FOXM1C could promote cell proliferation, migration and invasion in ovarian cancer cells. Although our findings showed that FOXM1C preferably increased cell proliferation while FOXM1B promoted cell migration/invasion, the simultaneous reduction of both FOXM1 isoforms using thiostrepton led to corresponding effects in both cell proliferation as well as cell migration/invasion. The effect of FOXM1 was further confirmed by examining the expressions of known markers in cell migration/invasion, *MMP-9* and *uPAR*, which are in line with the metastatic role of FOXM1 reported in other human cancers [9], [16], [32], [33]. Whether FOXM1 directly regulate these markers or other downstream effectors requires further investigation. Nevertheless, all of these observations suggest that the aberrant up-regulation of FOXM1 attributes the tumorigenic properties in high-grade ovarian cancer.

ERK is one of a crucial mediator in Raf/ERK/MAPK signaling axis maintaining diverse cellular function including cell proliferation, apoptosis as well as migration [34]. Previously, ERK has been shown to be constitutively active in ovarian cancer and our identification of high expression level of pERK in histological high-grade ovarian cancer and cell lines indicates its crucial role in causing aggressive tumorigenesis [35]. Intriguingly, we found that pERK and FOXM1 levels were tightly correlated during cancer progression, especially in high-grade ovarian cancer. We speculated that elevated FOXM1 expression is triggered by the constitutive activation of ERK, which leads to enhanced cell motility in ovarian cancer. As expected, their relationship was confirmed by manipulating the activity of ERK, which resulted in parallel changes of FOXM1 expression. In another study, motifs resembling the ERK target sequence have been found in FOXM1 and activation of ERK activity was demonstrated to induce the transactivating ability of FOXM1C [22]. Thus, suppressing ERK activity by U0126 would reduce FOXM1 phosphorylation which finally leads to inhibition of FOXM1 expression by disrupting its own positive feedback loop [36]. Though it gave us a clue that the transactivation of FOXM1 depends on phosphorylation by ERK at the post-transcriptional level, there is lack of reports regarding the functional attributes of activated ERK/FOXM1 signaling axis in ovarian cancer. In the wound healing study, in fact, the decrease in wound closure rate due to inhibited ERK activity and the reduction of FOXM1 expression upon U0126 treatment strongly suggests that FOXM is one of the major downstream effectors of ERK signaling. Moreover, thiostrepton and U0126 suppressed cell migration to a similar extent (40% as compared to 50%). Taken together, we believe that up-regulated ERK activity and the subsequent increase of FOXM1 expression contribute further induction of oncogenic behaviors, supporting that ERK/FOXM1 signaling axis facilitates cell migration in ovarian cancer.

Previous studies have demonstrated the promising effect of thiostrepton in impairing the tumorigenic behaviors mediated by FOXM1 [26], [37], [38]. Given the differential sensitivity of FOXM1 to down-regulation by thiostrepton treatment in cell lines with various status of p53, it is tempting to speculate the importance of p53 for the action of thiostrepton. Based on the fact that loss of p53 function is a common event in tumors and FOXM1 is negatively regulated by p53, we speculated that losing p53 expression may cause the deregulation of FOXM1 and thereby cause cellular catastrophe and even cancer. Here, the p53 deleted cell line SKOV3 showed no response of FOXM1 to various doses of thiostrepton as well as MEK inhibitor, U0126, while FOXM1 could be easily suppressed at multiple doses in OVCA433 and A2780cp which carry wild type and mutated p53 genes respectively. Previous studies showed that thiostrepton may elicit apoptotic effect on cancer cells by stabilizing p53 expression [39] and the up-regulation of p53 at higher dosage may further assist the suppression of FOXM1. Interestingly, thiostrepton or U0126 had no influence on SKOV3 carrying deleted p53 gene, whereas ectopic expression of p53 abolished the expression of FOXM1 at both RNA and protein level, consistent with other studies [28], [30]. This uncovers the requisite role of p53 in mediating the suppressive effect of thiostrepton on FOXM1 expression. Apart from impairing the cell migration/invasion ability, another group also showed that the use of thiostrepton could also increase apoptosis and proliferative arrest in human cancer cells which further underscores the therapeutic value of thiostrepton [26], [37], [38], [39], [40]. Collectively, our study supports that thiostrepton functions by suppressing FOXM1 expression via p53 to subsequently alleviate the tumorigenicity and up-regulating p53 to induce cell death.

In conclusion, our study revealed a link between ERK/FOXM1 signaling axis and the metastatic behavior of ovarian cancer cells. Moreover, we uncovered a critical role of p53 in mediating the action of thiostrepton. Hence, both U0126 and thiostrepton represent promising starting points to establish anti-cancer therapy in ovarian cancer through the repression of FOXM1.

## Materials and Methods

### Cell lines and cell culture

Five immortalized human ovarian surface epithelial cells were used: HOSE10-2, HOSE11-12, HOSE17-1, HOSE20-3 and HOSE21-3 (from Professor George Tsao, The University of Hong Kong). Nine ovarian cancer cell lines were used: A2780s A2780cp, OV2008, C13* (from Professor Benjamin Tsang, The University of Ottawa), OV420, OV429, OV433, OVCAR3 and SKOV3 (American Type Culture Collection, Rockville). All cell lines were incubated at 37°C in 5% CO_2_ in either minimum essential medium or Dulbecco's modified Eagle medium (Gibco-BRL, Gaithersburg) with 10% fetal bovine serum (Gibco) and 1% Penicillin-Streptomycin (Gibco).

### Plasmids and transfection

The lentiviral pCDH-FOXM1B and pCDH-FOXM1C plasmids were generated by subcloning FOXM1B and FOXM1C cDNAs from pcDNA3-FOXM1B and pcDNA3-FOXM1C plasmids into pCDH-MSCV-MCS-EF1-GFP-Puro lentiviral plasmid (SBI, Mountain View, CA) respectively. The pRcCMV-p53 plasmid was from Prof. Randy YC Poon (Section of Biochemistry and Cell Biology, Hong Kong University of Science and Technology, Hong Kong). LipofectAMINE™ 2000 transfection reagent (Invitrogen Life Technologies, Carlsbad, CA) was used for cell transfection according to the manufacturers' instruction. The pCDH-MSCV-MCS-EF1-GFP-Puro and pcDNA3.0 empty vectors were used as negative controls. To establish FOXM1B and FOXM1C stably expressing cells, lentiviral infected cells were selected by 2 µg/ml Puromycin (Sigma) for 2 weeks and verified by western blot analysis.

### Semi-quantitative (SQ-PCR) and Quantitative (QPCR) reverse transcriptase-polymerase chain reaction

Total RNA was isolated by TRIzol (Invitrogen) according to the manufacturer's protocol. Complementary DNA was synthesized using reverse transcription reagent kit (Applied Biosystems, Foster City). Subsequently, cDNA was used to perform PCR using specific genes primers of *FOXM1B* (forward, 5′-TTGCCCCCAAGGTGCTGCTA-3′ and reverse, 5′- GGAGATTGGGACGAATCCTC-3′), *FOXM1C* (forward, 5′-CACCCATCACCAGCTTGTTT3′ and reverse, 5′-GGAGATTGGGACGAATCCTC-3′), *MMP9* (forward, 5′- CATCGTCATCCAGTTTGGTG and reverse, 5′-GCCTTGGAAGATGAATGGAA-3′) and *uPAR* (forward, 5′-GCCTTACCGAGGTTGTGTGT-3′ and reverse, 5′- GGCAGATTTTCAAGCTCCAG -3′) under the condition of 94°C (5 min), followed by 30–35 cycles of 95°C (30 sec), 57°C (30 sec) and 72°C (30 sec), then finally 72°C (10 min) and 4°C. The relative amount of gene was normalized against GAPDH mRNA.

Q-PCR was performed by TaqMan® Gene Expression Assays and SYBR Green Gene Expression Assay. To validate the expression of *FOXM1* isoforms, specific primers targeting against *FOXM1B* and *FOXM1C* were designed according to Kalin *et al* [11] and purchased from Applied Biosystems. To examine the expression of p53, specific TaqMan probes (Applied Biosystems) included in PCR mix were subjected to PCR under the condition of 95°C for 2 minutes, 40°C cycles at 95°C for 15 seconds and 60°C for 1 minute. Relative amount of RNA in each sample was determined by comparative CT method with the 7500 System SDS software (version 1.3.1), from which the fold difference was compared to the internal control *18S* and *TBP* and the relative fold difference was then normalized to the calibrator. The relative expression level of target gene was interpreted as the fold difference to the endogenous control.

### Western blotting and immunohistochemical (IHC) analyses

Total protein was prepared by adding suitable amount of 1x lysis buffer to the cell pellet. Protein samples were separated by SDS-PAGE according to the standard western blot procedures and then electroblotted to the Hybond-P membranes (Amersham Pharmacia Biotech, Cleveland) followed by blocking with 5% skim milk for 30 minutes. The blots were incubated with primary monoclonal antibodies; anti-ERK, anti-pERK (Cell Signaling Technology), anti-FOXM1 (K-19), anti-p53 (Sigma Chemical Co., St. Louis, MO) and anti-β-actin (Sigma-Aldrich, MO) overnight at 4°C. Anti-mouse or anti-rabbit secondary antibody conjugated to horse radish peroxide (Amersham Pharmacia Biotech, OH) was used and the signal was then visualized by enhanced chemiluminescence (Amersham).

For immunohistochemical analysis, formalin-fixed paraffin-embedded ovarian cancer tissue array (OVC1021) (Pantomics Inc, San Francisco, CA) was immuno-stained with primary monoclonal anti-FOXM1 (C-20, Santa Cruz) or pERK (Cell Signaling Technology) in 1∶50 dilution. All of the stained tissue sections were identified as either positive or negative. For those positive immuno-reactive samples, the staining was scored in terms of proportion of stained area (0–100%) and intensity (+1, faint; +2, moderate; +3, strong and +4, very strong). The immunoreactivity was determined by multiplying the percentage of stained area and intensity. All tissue section was examined and scored by two investigators.

### Cell viability assay

Cell proliferation kit (XTT) (Roche) was used to measured cell viability in the time course of 4 days according to the manufacture's manual. Three independent experiments were performed in triplicate.

### Wound healing assay

For wound healing assay, cells were seeded to full confluence in a monolayer in a six-well plate. A micro-pipette tip was used to scratch a single wound in each well. The plate was incubated at 5% CO_2_ at 37°C. Images were taken at 0, 12 and 24 hours after scratching and were subsequently analyzed. The average cell migration rate was expressed as relative width of wound/time. Three independent experiments were carried out in duplicate.

### Cell migration assay

For quantifying the cell migration ability using colorimetric cell migration assay, a cell suspension containing 1.5×10^5^ cells in serum free medium was added to each insert (Chemicon International) while 500 µl serum-containing medium as a chemo-attractant was added to the lower chamber (for FOXM1 inhibition test, 10 µM of thiostrepton was added included). The plate was incubated for 12 hours at 37°C at 5% CO_2_ allowing the cells to pass through the pores in the membrane. Afterwards, remaining cell suspension was removed and the inserts were stained with Cell Stain. Three independent sections in each transwell filter were visualized under the microscope (TE300, Nikon) and the number of cells was counted. The experiments were performed thrice.

### Cell invasion assay

BD BioCoat™ Matrigel™ Invasion Chamber (BD Bioscience) was used to study the cell invasion. Inserts and wells were rehydrated before the start of experiment. Cell suspension of 5×10^5^ cells in serum free medium was added to each insert while 500 µl serum-containing medium as a chemo-attractant was added to the lower chamber (for FOXM1 inhibition test, 10 µM of thiostrepton was added to both serum free and serum containing medium). The plate was incubated for 36–48 hours at 37°C at 5% CO_2_ allowing the cells to pass through the pores on the membrane. Afterwards, remaining cell suspension was removed and the inserts were stained with 500 µl of Crystal Violet. Three independent sections in each transwell filter were visualized under the microscope (TE300, Nikon) and the number of cells was counted. The experiments were performed thrice.

### Data analysis

Student's t test was applied to analyze parametric data while Mann-Whitney test was used for non-parametric data. Pearson Chi-Square test was applied to examine the correlation between FOXM1 and clinical parameters. A *P*-value less than 0.05 was considered as significant in all tests. All data were expressed as mean ± SD.

## Supporting Information

Figure S1 **XTT assay showed that no significant change in the cell proliferation rate of OVCA433 by treatment of thiostrepton in a lower dose (5 and 10 µM) within 3 days.**
(TIF)

Figure S2 **Wound healing assay showed no influence in cell migration of SKOV3 upon treatment with 5 µM and 10 µM of thiostrepton for 12–24 hrs when compared to untreated control.**
(TIF)

Figure S3 **(** ***Upper*** **) Western blot analysis showed that no change in FOXM1 expression in SKOV3 upon treatment of 10 µM U0126 for 4 to 8 Hrs. (** *Lower*) Wound healing assay demonstrated that no change in cell migration of SKOV3 upon treatment with 10 µM U0126 for 12–24 hrs when compared to untreated control.
(TIF)
