# Supplementary figures and images for: Aberrant Activation of ERK/FOXM1 Signaling Cascade Triggers the Cell Migration/Invasion in Ovarian Cancer Cells
Source: PLoS One. 2011 Aug 17;6(8):e23790. doi: 10.1371/journal.pone.0023790 (PMC3157468; doi:10.1371/journal.pone.0023790)

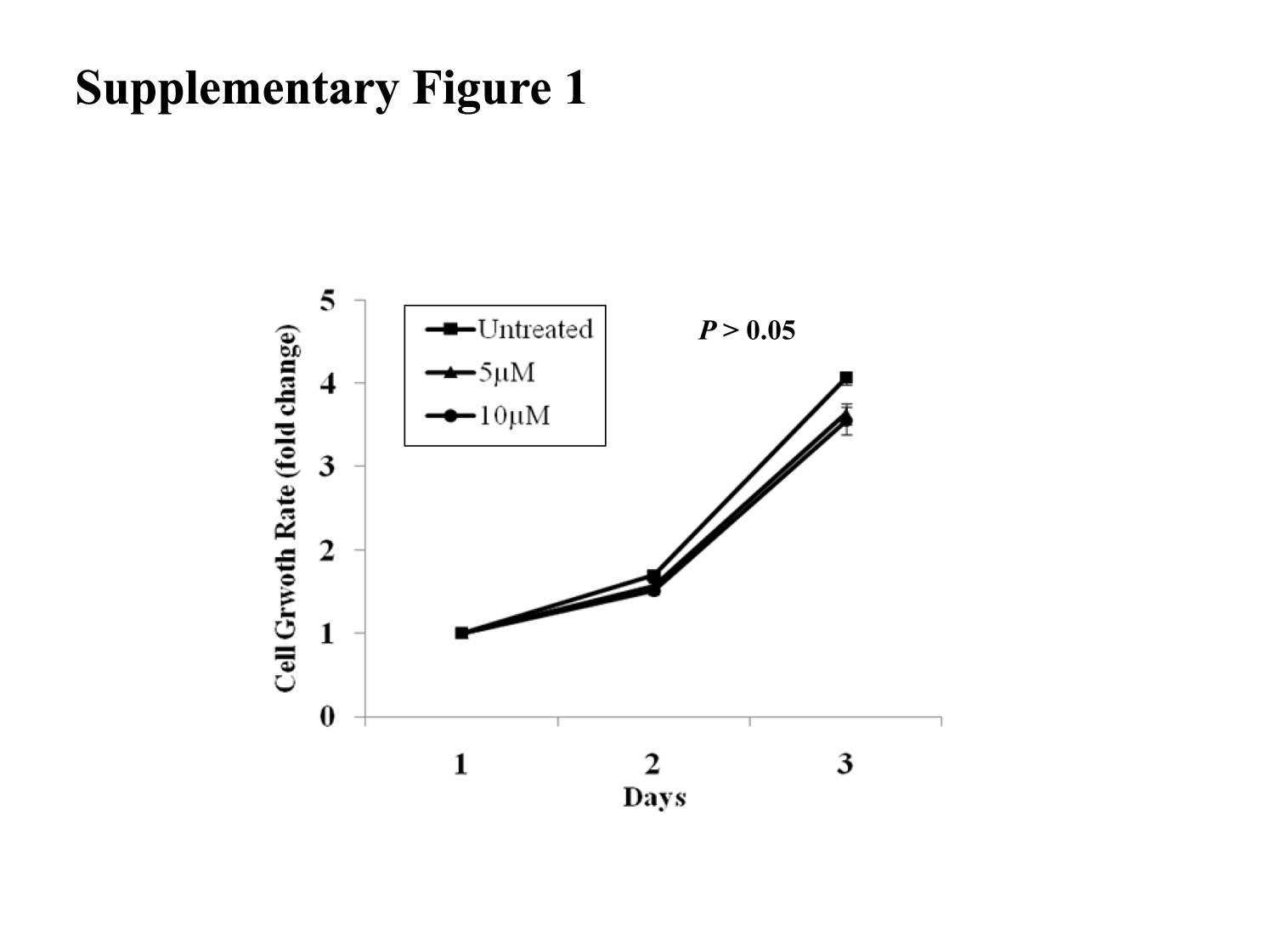

Supplement: Figure S1 — XTT assay showed that no significant change in the cell proliferation rate of OVCA433 by treatment of thiostrepton in a lower dose (5 and 10 µM) within 3 days. (TIF) [file pone.0023790.s001.tif]

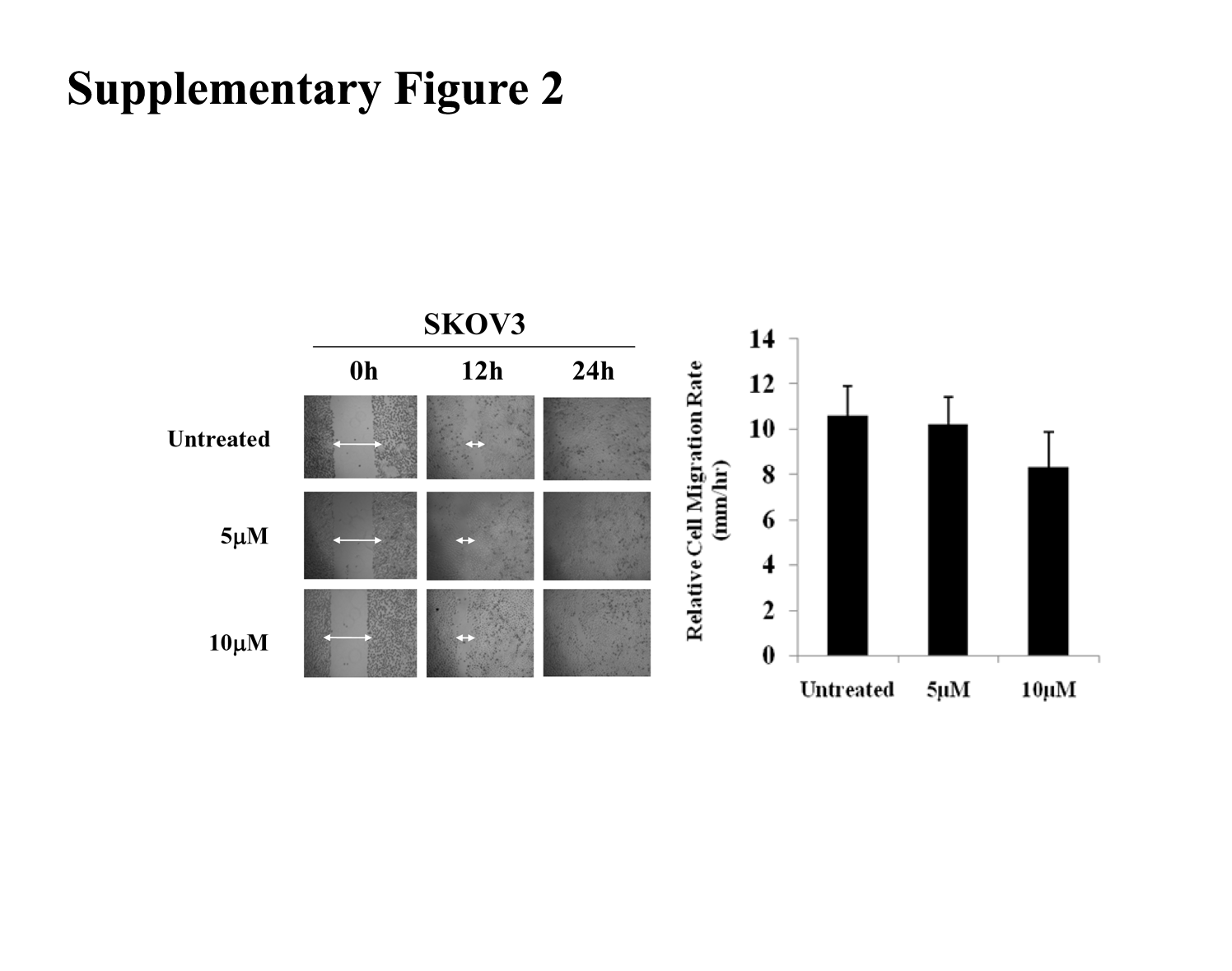

Supplement: Figure S2 — Wound healing assay showed no influence in cell migration of SKOV3 upon treatment with 5 µM and 10 µM of thiostrepton for 12–24 hrs when compared to untreated control. (TIF) [file pone.0023790.s002.tif]

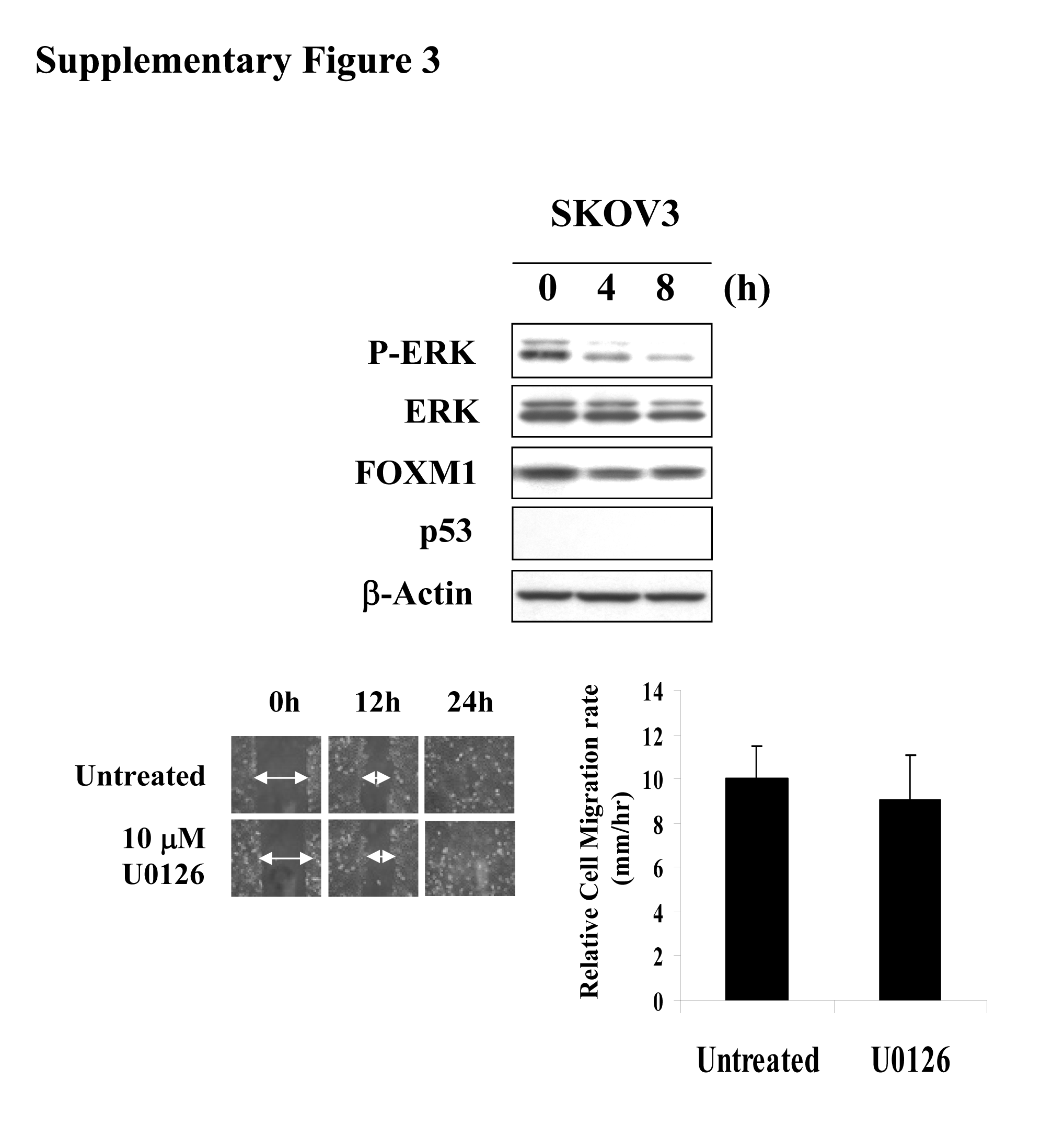

Supplement: Figure S3 — ( Upper ) Western blot analysis showed that no change in FOXM1 expression in SKOV3 upon treatment of 10 µM U0126 for 4 to 8 Hrs. ( Lower) Wound healing assay demonstrated that no change in cell migration of SKOV3 upon treatment with 10 µM U0126 for 12–24 hrs when compared to untreated control. (TIF) [file pone.0023790.s003.tif]
